# Good reasons to vaccinate: mandatory or payment for risk?

**DOI:** 10.1136/medethics-2020-106821

**Published:** 2020-11-05

**Authors:** Julian Savulescu

**Affiliations:** 1 Faculty of Philosophy, University of Oxford, Oxford, UK; 2 Murdoch Childrens Research Institute, Parkville, Victoria, Australia; 3 Melbourne Law School, University of Melbourne, Melbourne, Victoria, Australia

**Keywords:** behaviour modification, coercion, technology/risk assessment, philosophical ethics, public health ethics

## Abstract

Mandatory vaccination, including for COVID-19, can be ethically justified if the threat to public health is grave, the confidence in safety and effectiveness is high, the expected utility of mandatory vaccination is greater than the alternatives, and the penalties or costs for non-compliance are proportionate. I describe an algorithm for justified mandatory vaccination. Penalties or costs could include withholding of benefits, imposition of fines, provision of community service or loss of freedoms. I argue that under conditions of risk or perceived risk of a novel vaccination, a system of payment for risk in vaccination may be superior. I defend a payment model against various objections, including that it constitutes coercion and undermines solidarity. I argue that payment can be in cash or in kind, and opportunity for altruistic vaccinations can be preserved by offering people who have been vaccinated the opportunity to donate any cash payment back to the health service.

## Introduction

We are in the midst of a global pandemic with COVID-19 and there is a race to develop a vaccine. At the time of writing, there are 53 vaccines in clinical trials on humans (plus five that have bypassed the full trial process) and at least 92 preclinical vaccines under active investigation in animals. There are a number of different approaches: (1) genetic—using mRNA to cause the body to produce viral proteins; (2) viral vector—using genetically modified viruses such as adenovirus to carry sections of coronavirus genetic material; (3) protein—delivering viral proteins (but not genetic material) to provoke an immune response; (4) inactivated or attenuated coronavirus; (5) repurposing existing vaccines, eg, BCG (bacillus Calmette–Guérin).^1^

Given the mounting number of deaths globally, and the apparent failure of many countries to contain the pandemic without severely damaging or problematic lockdowns and other measures, there have been calls to make a vaccine, if it were approved, mandatory.^2^ Mandatory vaccination has not been ruled out within the UK.^3^

The first part of this article asks when, if ever, a vaccine should be mandatory. I will create a set of criteria and a decision algorithm for mandatory vaccination. I will argue that in the case of COVID-19, some of these criteria may not be satisfied. The second part of the article argues that in the case of COVID-19, it may be ethically preferable to incentivise vaccine uptake. I will justify incentivisation and discuss different kinds of incentives.

## Ethics of mandatory COVID-19 vaccination

There is a large body of literature on the justification for the use of coercion in public health and infectious disease in particular. Mandatory vaccination is typically justified on Millian grounds: harm to others. According to John Stuart Mill, the sole ground for the use of state coercion (and restriction of liberty) is when one individual risks harming others.^4^ The most prominent arguments from bioethicists appeal to preventing harm to others.^5–7^ In the case of children, significant risk of harm to the child is also a ground for state protection. Bambery *et al* 8 give the example of a child taking a box of toxic bleach to school, potentially harming himself and other children. Teachers are entitled to restrain the child and remove the poison both because of risk to the child and to other children.^8^ Flanigan uses a similar example of a person shooting a gun into a crowd.^5^

The Nuffield Council of Bioethics produced an influential report on public health which considers when coercion and mandatory vaccination might be justified:

When assessing whether more directive policies are acceptable, the following factors should be taken into account: the risks associated with the vaccination and with the disease itself, and the seriousness of the threat of the disease to the population. In the case of incentivised policies, the size of the incentive involved should be appropriate so that it would not unduly compromise the voluntariness of consent.

We identified two circumstances in which quasi-mandatory vaccination measures are more likely to be justified. First, for highly contagious and serious diseases, for example with characteristics similar to smallpox. Second, for disease eradication if the disease is serious and if eradication is within reach.^9^

I will elaborate on these brief suggestions and provide a novel structured algorithm for when vaccination should be mandatory.

COVID-19 is almost unique because of the gravity of the problem at the global level: not only is there cost in terms of lives from COVID-19, there is also the extraordinary economic, health and well-being consequences of various virus-control measures, including lockdown, which will extend into the future. Probably never before has a vaccine been developed so rapidly and the pressure to use it so great, at least at the global level.

There is a strong case for making any vaccination mandatory (or compulsory) if four conditions are met:

1. There is a grave threat to public health
2. The vaccine is safe and effective
3. Mandatory vaccination has a superior cost/benefit profile compared with other alternatives
4. The level of coercion is proportionate.

Each of these conditions involves value judgements.

### Grave threat to public health

So far, there have been over 1 million deaths attributed to COVID-19 globally (as of 30 September 2020).^10^ In the UK alone, it was predicted in influential early modelling that 500 000 would have died if nothing was done to prevent its spread. Even with the subsequent introduction of a range of highly restrictive lockdown measures (measures which could themselves come at a cost of 200 000 non-COVID-19 lives according to a recent UK government report),^11^ more than 42 000 (as of 30 September 2020)^12^ have died in the UK within 28 days of a positive test.

The case fatality rate was originally estimated to be as high as 11%, but (as is typical with new diseases) this was quickly scaled down to 1.5% or even lower.^13^ The infection fatality rate (IFR, which accounts for asymptomatic and undiagnosed cases) is lower still as it has become clear that there are a large number of asymptomatic and mild cases. For example, the Centre for Evidence Based Medicine reports that “In Iceland, where the most testing per capita has occurred, the IFR lies somewhere between 0.03% and 0.28%”.^14^

Of course, how you define “grave” is a value judgement. One of the worst-affected countries in the world in terms of COVID-19-attributed deaths per million is Belgium. The mortality is (at the time of writing) around 877 per million population, which is still under 0.1%, and the average age of death is 80. Of course, Belgium and most other countries have taken strict measures to control the virus and so we are not seeing the greatest possible impact the virus could have. Yet others such as Brazil and Sweden have intervened to a much lesser degree, yet (currently) have rates of 687 and 578 deaths per million respectively. Sweden’s April all-cause deaths and death rate at the peak of its pandemic so far, while extremely high, were surpassed by months in 1993 and 2000.^15^

The data are complex and difficult to compare with different testing rates, and ways of assigning deaths and collecting data differing from country to country. For example, Belgium counts deaths in care homes where there is a suspicion that COVID-19 was the cause (without the need for a positive test) and, until recently, the UK counted a death which followed any time from a COVID-19 positive test as a COVID-19 death. Moreover, there have been huge behavioural changes even in countries without legally enforced lockdowns. Furthermore, the IFR varies wildly by age-group and other factors. Even among survivors, there is emerging evidence that there may be long-term consequences for those who have been infected but survived. Long COVID-19 health implications may present a grave future public health problem. Nevertheless, some might still argue that this disease has not entered the “grave” range that would warrant mandatory vaccination. The Spanish influenza killed many more (50–100 million),^16^ and it afflicted younger rather than older people, meaning even more “life years” were lost. It is not difficult to imagine a Superflu, or bioengineered bug, which killed 10% across all ages. This would certainly be a grave public health emergency where it is likely mandatory vaccination would be employed.

Deciding whether COVID-19 is sufficiently grave requires both more data than we have and also a value judgement over the gravity that would warrant this kind of intervention. But let us grant for the sake of argument that COVID-19 *is* a grave public health emergency.

### Vaccine is safe and effective

There are concerns that testing has been rushed and the vaccine may not be safe or effective.^17^

First, although the technology being used in many of these vaccine candidates has been successfully used in other vaccines, no country has ever produced a safe and effective vaccine against a coronavirus. So in one way, we are all in uncharted waters.

Second, any vaccine development will be accelerated in the context of a grave public health emergency. The inherent probabilistic nature of the development of any biologic means that no vaccine could be said to be 100% safe. There will be risks and those risks are likely to be greater than with well-established vaccines.

Thirdly, some side effects may take time to manifest.

This is not to support the anti-vaccination movement. Vaccines are one of the greatest medical accomplishments and a cornerstone of public health. There are robust testing procedures in place in most jurisdictions to ensure that licensed COVID-19 vaccines are both effective and safe. It is only to acknowledge that everything, including vaccination, has risks. Perhaps the biggest challenge in the development of a vaccine for COVID-19 will be to be honest about the extent of those risks and convey the limitations of confidence in safety and efficacy relative to the evidence accrued.

There is an ethical balance to be struck: introducing a vaccine early and saving more lives from COVID-19, but risking side effects or ineffectiveness versus engaging in longer and more rigorous testing, and having more confidence in safety and efficacy, but more people dying of COVID-19 while such testing occurs. There is no magic answer and, given the economic, social and health catastrophe of various anti-COVID-19 measures such as lockdown, there will be considerable pressure to introduce a vaccine earlier.

To be maximally effective, particularly in protecting the most vulnerable in the population, vaccination would need to achieve herd immunity (the exact percentage of the population that would need to be immune for herd immunity to be reached depends on various factors, but current estimates range up to 82% of the population).^18^

There are huge logistical issues around finding a vaccine, proving it to be safe, and then producing and administering it to the world’s population. Even if those issues are resolved, the pandemic has come at a time where there is another growing problem in public health: vaccine hesitancy.

US polls “suggest only 3 in 4 people would get vaccinated if a COVID-19 vaccine were available, and only 30% would want to receive the vaccine soon after it becomes available.”^18^

Indeed, vaccine refusal appears to be going up. A recent Pew survey suggested 49% of adults in the USA would refuse a COVID-19 vaccine in September 2020.^19^

If these results prove accurate then even if a safe and effective vaccine is produced, at best, herd immunity will be significantly delayed by vaccine hesitancy at a cost both to lives and to the resumption of normal life, and at worst, it may never be achieved.

There remain some community concerns about the safety of all pre-existing vaccines, including many that have been rigorously tested and employed for years.

In the case of COVID-19, the hesitancy may be exacerbated by the accelerated testing and approval process which applies not only to Sputnik V (the controversial “Russian vaccine”). Speaking about America’s vaccine programme, Warp Speed, Donald Trump applauded its unprecedented pace:

…my administration cut through every piece of red tape to achieve the fastest-ever, by far, launch of a vaccine trial for this new virus, this very vicious virus. And I want to thank all of the doctors and scientists and researchers involved because they’ve never moved like this, or never even close.^20^

The large impact on society means the vaccine will be put to market much more quickly than usual, perhaps employing challenge studies or other innovative designs, or by condensing or running certain non-safety critical parts of the process in parallel (for example, creating candidate vaccines before its approval).

While the speed is welcomed by politicians and some members of the public, the pressure to produce a candidate vaccine, and the speed at which it has been done, may be also perceived (perhaps unfairly) to increase the likelihood of the kind of concerns that lead to vaccine hesitancy: concerns over side-effects that are unexpected or rare, or that take longer to appear than the testing process allows for, or that for another reason may be missed in the testing process.

The job to be done will not only be to prove scientifically that the vaccine is safe and effective, but also to inform and reassure the public, especially the group who are willing to take the vaccine in theory—but only after others have tried it out first.

The question remains of how safe is safe enough to warrant mandatory vaccination. It is vanishingly unlikely that there will be absolutely no risk of harm from any biomedical intervention, and the disease itself has dramatically different risk profiles in different groups of the population. In an ideal world, the vaccine would be proven to be 100% safe. But there will likely be some risk remaining. Any mandatory vaccination programme would therefore need to make a value judgement about what level of safety and what level of certainty are safe and certain enough. Of course, it would need to be very high, but a 0% risk option is very unlikely.

A COVID-19 vaccine may be effective in reducing community spread and/or preventing disease in individuals. Mandatory vaccination is most justifiable when there are benefits to both the individual and in terms of preventing transmission. If the benefits are only to individual adults, it is more difficult to support mandatory vaccination. One justification would be to prevent exhaustion of healthcare services in an emergency (eg, running out of ventilators), which has been used a basis of restriction of liberty (it was the main justification for lockdown). It could also be justified in the case of protection of children and others who cannot decide for themselves, and of other adults who either cannot be vaccinated for medical reasons.

### Better than the alternatives

**Figure 1:**
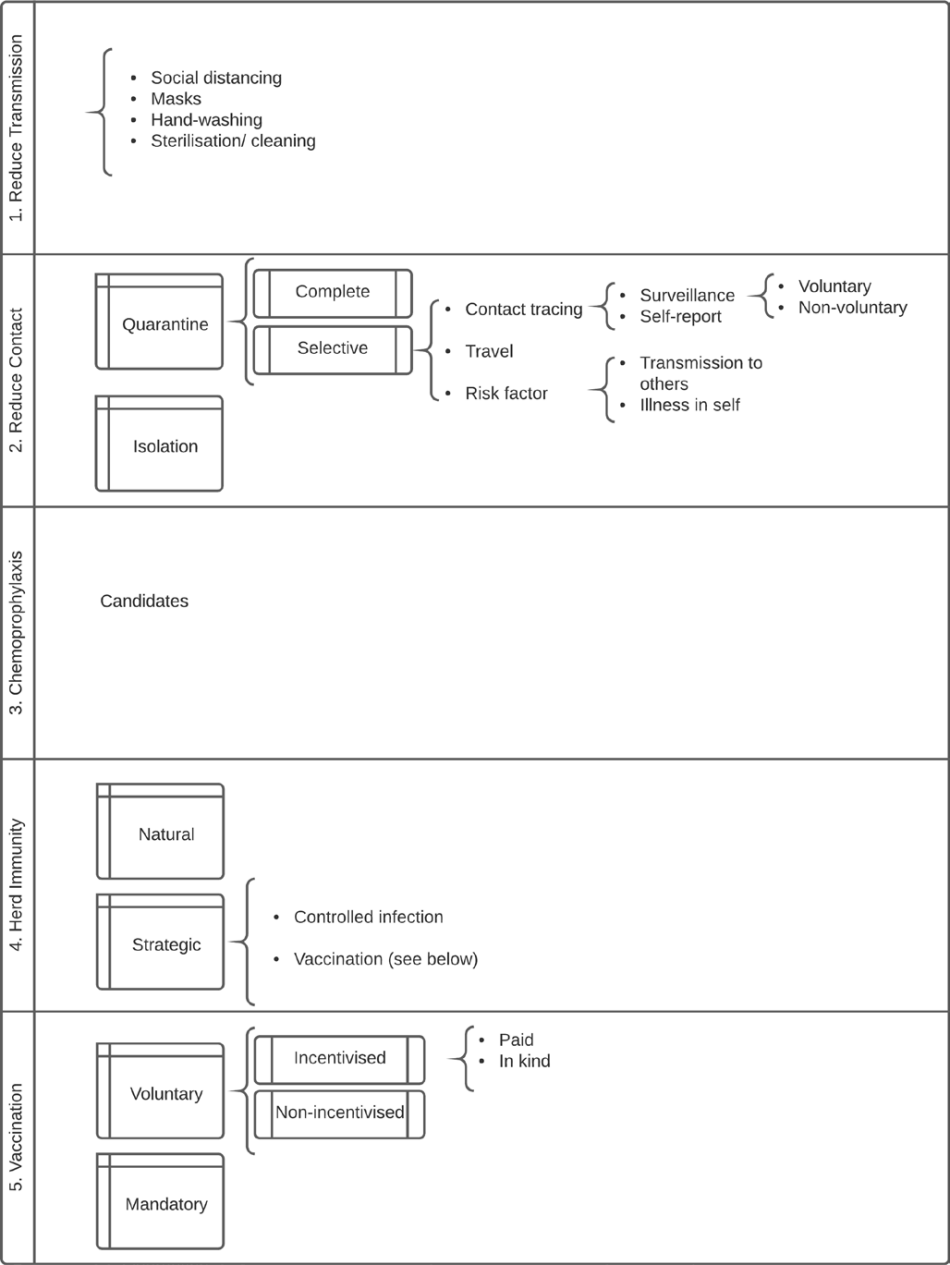
Strategies for prevention of infectious disease.

It is a standard principle of decision theory that the expected utility of a proposed option must be compared with the expected utility of relevant alternatives. There are many alternatives to mandatory vaccination. See figure 1 for a summary of the range of strategies for preventing infectious disease.

A popular position, especially among medical professionals,^7^ is that we don’t need mandatory vaccination because people are self-interested or altruistic enough to come forward for vaccination. We can reach herd immunity without mandatory vaccination.

If this were true, all well and good, but the surveys mentioned above cast doubt on this claim with regard to the future COVID-19 vaccine. Moreover, reaching herd immunity is not good enough.

First, how fast we reach herd immunity is also important. In a pandemic, time is lives. If it takes a year to reach herd immunity, that could be thousands or tens of thousands of lives in one country.

Second, herd immunity is necessary because some people cannot be vaccinated for medical reasons: they have allergies, immune problems, or other illnesses. The elderly often don’t mount a strong immune response (that is why it is better to vaccinate children for influenza because they are the biggest spreaders of that disease^7^—although COVID-19 appears to be different on the current evidence). And immunity wanes over time—so even people previously vaccinated may become vulnerable.

Even when national herd immunity is achieved, local areas can fall below that level over time, causing outbreaks, as happened with measles recently. This is especially likely to happen where people opposed to vaccines tend to cluster toghether—for example, in the case of certain religious communities. So ideally we need better than herd immunity to ensure that people are protected both over time and in every place.

These are thus reasons to doubt whether a policy of voluntary vaccination will be good enough, though it remains to be seen.

There are other policies that might obviate the need for mandatory vaccination. South Korea has kept deaths down to about 300 (at the time of writing) with a population of 60 000 000 with a vigorous track and trace programme (although it was criticised for exposing extra-marital affairs and other stigmatised behaviours).^21^ Other countries have enforced quarantine with tracking devices. There could be selective lockdown of certain groups,^22^ or for intermittent periods of time.

The long-term costs and benefits of such policies would have to be evaluated. That is, we should calculate the expected utility of mandatory vaccination (in combination with other policies) and compare it to alternative strategies (or some other combination of these). How utility should be evaluated is an ethical question. Should we count deaths averted (no matter how old), life years lost or lost well-being (perhaps measured by quality adjusted life years)?^23^ Should we count loss of liberty or privacy into the other side the equation?

It may be that a one-off mandatory vaccination is a significantly smaller loss of well-being or liberty than these other complex resource intensive strategies.

So we cannot say whether a mandatory policy of COVID-19 vaccination is ethically justified until we can assess the nature of the vaccine, the gravity of the problem and the likely costs/benefit of alternatives. But it is certainly feasible that it could be justified.

It is important to recognise that coercive vaccination *can* be justified. This is easy to see by comparing it to other coercive interventions in the public interest.

#### Conscription in war

In the gravest emergencies, where the existence and freedom of the whole population is at stake, people are conscripted to serve their country, often with high risk of death or permanent injury. We often analogise the pandemic to a war: we are fighting the virus. If people can be sent to war against their will, in certain circumstances some levels of coercion are justified in the war on the virus. Notably, in conditions of extreme emergency in past wars (graver than currently exist for COVID-19), imprisonment or compulsion have even been employed.^24^

#### Taxes

A more mundane example is the payment of taxes. Taxes benefit individuals because tax revenue allows the preservation of public goods. But if sufficient numbers of others are paying their taxes, it is in a person’s self-interest to free ride and avoid taxes. Indeed, paying taxes may result in harm in some circumstances.^24^ In the USA, where there is a large private healthcare sector, paying your taxes may mean you cannot pay for lifesaving medical care that you would otherwise have been able to afford. Still, taxes are mandatory based on considerations of fairness and utility.

#### Seat belts

Seat belts are mandatory in the UK and many other countries, whereas they were previously voluntary. Interestingly, 50% or so of Americans initially opposed making seat belts mandatory, but now 70% believe mandatory laws are justified.^25^

Seat belts reduce the chance of death if you are involved in a car accident by 50%. They are very safe and effective. Notably, they do cause injuries (seat belt syndrome) and even, very occasionally, death. But the chances of being benefitted by wearing them vastly outweigh these risks, so they are mandatory, with enforcement through fines. I have previously likened vaccination to wearing a seat belt.^25^

#### Pre-existing mandatory vaccination

Mandatory vaccination policies are already in place in different parts of the world. Mandatory vaccination policies are those that include a non-voluntary element to vaccine consent and impose a penalty or cost for unjustified refusal (justified refusal includes those who have a contraindicating medical condition, or those who already have natural immunity). There are a range of possible penalties or costs which can coerce people. Australia has the “No Jab, No Pay” scheme which withholds child benefits if the child is not vaccinated, and a “No Jab, No Play” scheme which withholds kindergarten childcare benefits. Italy introduced fines for unvaccinated children who attend school. In the USA, state regulations mandate that children cannot attend school if they are not vaccinated, and healthcare workers are required to vaccinate. Some US states (eg, Michigan) make exemptions difficult to obtain by requiring parents to attend immunisation education courses^26^ (see also^27 28^).

**Figure 2:**
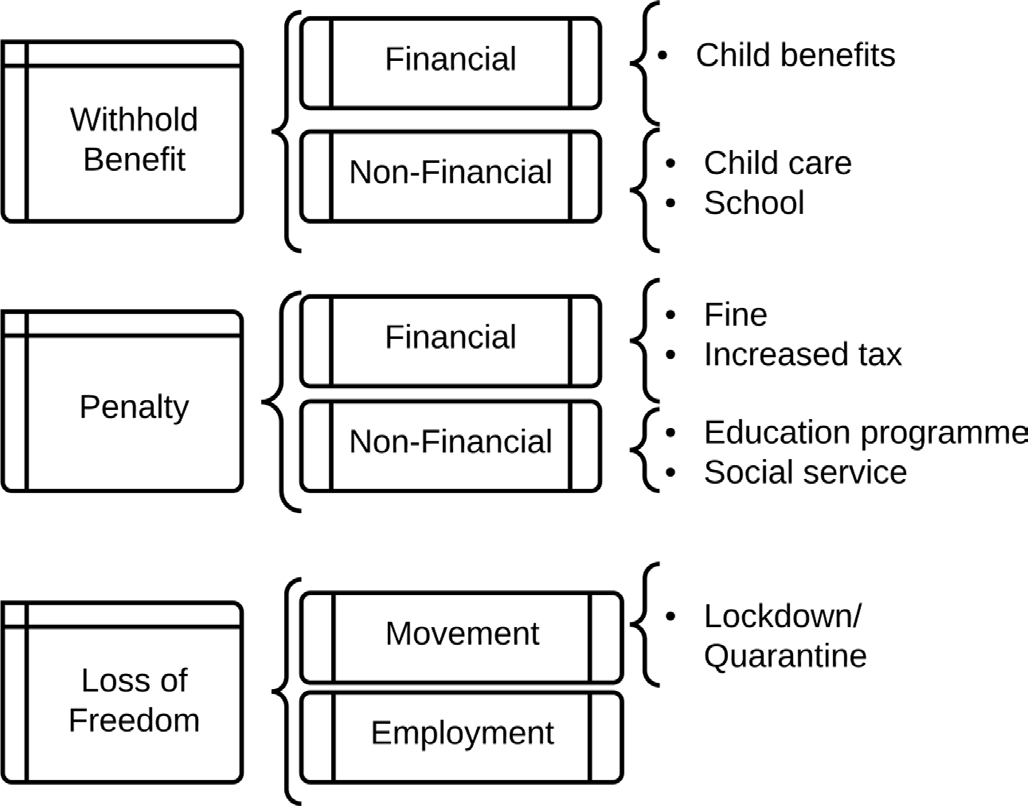
Cost of mandatory/coercive vaccination.

Figure 2 summarises the range of coercive policies that can constitute mandatory vaccination.

### Coercion is proportionate

In public health ethics, there is a familiar concept of the “least restrictive alternative”.^28^ The least restrictive alternative is the option which achieves a given outcome with the least coercion (and least restriction of liberty).

This is a very weak principle: it uses liberty as tie breaker between options with the same expected utility. More commonly, however, we need to weigh utility against liberty. That is, a more restrictive policy will achieve more expected utility—but is it justified?

According to a principle of proportionality, the additional coercion or infringement in liberty is justified if it is proportionate to the gain in expected utility of the more coercive intervention compared with next best option. That is, additional coercion is justified when the restriction of liberty is both minimised and proportionate to the expected advantages offered by the more coercive policy.

As we can see from the previous section and figure 2, there are a variety of coercive measures. (The Nuffield Council has created a related “Intervention Ladder”,^29^ though this includes education and incentives, as well as coercive measures.) Penalties can be high. In war, those who conscientiously objected to fighting went to jail or were forced to perform community service (or participate in medical research). In France, parents were given a suspended prison sentence for refusing to vaccinate their child.^30^

While there are legitimate concerns that the effectiveness of these policies in different contexts has been inadequately investigated, a number of these policies have been shown to increase vaccination rates.^31^

Notably, the fine or punishment for avoiding taxes varies according to the gravity of the offence. The fine for not wearing a seat belt is typically small. A modest penalty for not being vaccinated in a grave public health emergency could be justifiable. For example, a fine or restriction of movement might be justified.

**Figure 3:**
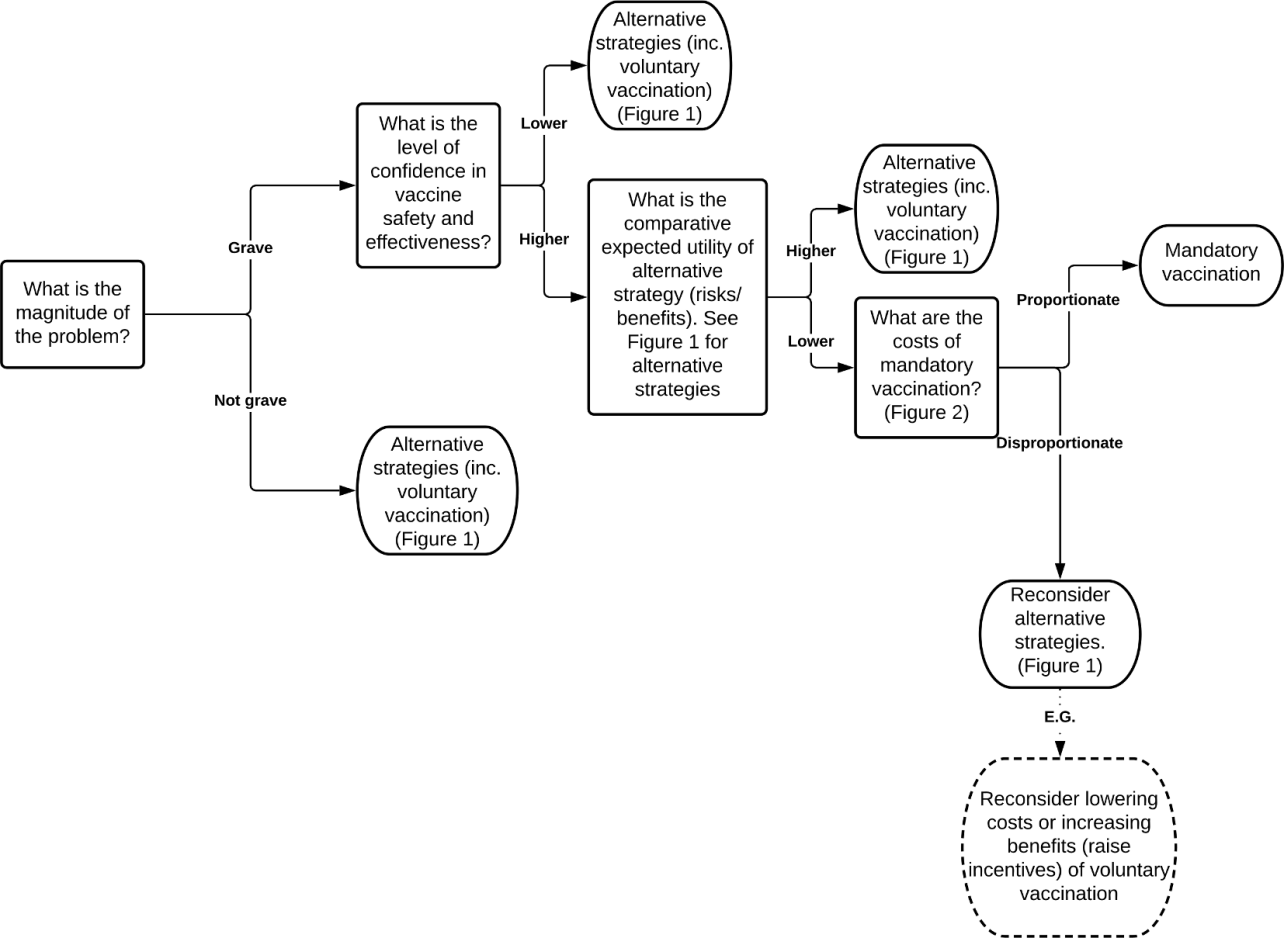
Algorithm for mandatory vaccination.

Figure 3 combines these four factors into an algorithm for justified mandatory vaccination.

These four factors can be justified in several ways. They represent a distillation and development of existing principles in public health ethics, for example, the least restrictive alternative. They can also be justified by the four principles of biomedical ethics.

For example, justice is about the distribution of benefits and burdens across a population in a fair manner. Justice and beneficence, in the context of vaccination policies, both require that the problem addressed is significant and vaccination is an effective means of addressing it. Non-maleficence requires that the risk imposed on individuals be small. Respect for autonomy and justice both require that coercion be applied only if necessary and that it be proportionate to additional utility of mandatory vaccination (and that such coercion be minimised, which is a feature of proportionality).

It is important to recognise that vaccines may have benefits both to the individual and to others (the community). If the vaccine has an overall net expected utility for the individual, beneficence supports its administration.

How great a sacrifice (loss of liberty or risk) can be justified? The most plausible account is provided by a duty of easy rescue: when the cost to an individual is small of some act, but the benefit or harm to another is large, then there is a moral obligation to perform that act. I have elsewhere argued for a collective duty of easy rescue: where the cost of some act to an individual is small, and the benefit of everyone doing that act to the collective is large, there is a collective duty of easy rescue.^32^ Such a principle appropriately balances respect for autonomy with justice.

Whether mandatory vaccination for any disease can be justified will depend on precise facts around the magnitude of the problem, the nature of the disease and vaccination, the availability and effectiveness of alternative strategies and the level of coercion. Elsewhere I compare mandatory vaccination for influenza and COVID-19 in more detail.^27^

## Better than coercion? Payment for risk

Given the risks, or perceived risks, of a novel COVID-19 vaccine, it would be practically and perhaps ethically problematic to introduce a mandatory policy, at least initially (when uncertainty around safety will be greater). Is there a more attractive alternative?

The arguments in favour of vaccination, particularly for those at lower risk (children, young people and those previously infected) can be based on a principle of solidarity. After all, “We are in this together” has been a recurrent slogan supporting pandemic measures in different countries. Those at low risk are asked to do their duty to their fellow citizens, which is a kind of community service. Yet they have little to personally gain from vaccination. The risk/benefit profile looms large for those at lowest risk.

However, another way of looking at this is that those at low risk are being asked to do a job which entails some risk., so they should be paid for the risk they are taking for the sake of providing a public good. And although it may be unlikely to influence so-called 'anti-vaxxers', it may influence a good portion of the 60% of American adults who responded in a March 2020 poll that they would either delay vaccination or didn’t know about vaccination.^33^

I have previously argued that we should reconceive live organ donation and participation in risky research, including challenge studies,^34^ as jobs where risk should be remunerated, much like we pay construction workers and other dangerous professions both for the job and for the risk involved.^35 36^ While the risk profile for approved vaccinations means that it differs from these examples, it could be compared to a job such as social work as a further argument in favour of payment. People may legitimately be incentivised to take on risks, as the Nuffield Council recognises in its Intervention Ladder.^29^

The advantage of payment for risk is that people are choosing voluntarily to take it on. As long as we are accurate in conveying the limitations in our confidence about the risks and benefits of a vaccine, then it is up to individuals to judge whether they are worth payment.

Of course, that is a big ask. It would require government to be transparent, explicit and comprehensive in disclosure of data, what should be inferred and the limitations on the data and confidence. This has often not been the case—one only has to remember the denial of the risks of bovine spongiform encephalopathy (BSE) at the height of the crisis by the British government, when in 1990 the Minister for Agriculture, Fisheries and Food, John Gummer proudly fed his 4-year-old daughter, Cordelia, a hamburger in front of the world’s media, declaring British beef safe. (Gummer was awarded a peerage in 2010 and is now Lord Deben.)^37^

There is also a danger that payment might signal lack of confidence in safety. That is a real risk and one that I will address in the “payment in kind” section below.

But the basic ethical point (public acceptability aside) is that, if a vaccine is judged to be safe enough to be used without payment, then it is safe enough to be used with payment.^36^ Payment itself does not make a vaccine riskier. If a vaccine is considered too risky to be administered to the population, then it should not be administered, no matter whether coercively, through incentives, or through some other policy.

### Coercion?

A standard objection to payment for risk (whether it is risky research or live organ donation) is that it is coercive: it forces people to take risks against their better judgement. In Macklin’s words:

The reason for holding that it is ethically inappropriate to pay patients to be research subjects is that it is likely to be coercive, violating the ethical requirement that participation in research should be fully voluntary.^38^

As I have previously argued,^39^ this demonstrates deep conceptual confusion. Coercion exists when an option which is either desired or good is removed or made very unappealing. The standard example is a robber who demands “Your money or your life”. This removes the most desired and best option: your money and your life. The Australian “No Jab, No Pay”scheme arguably does constitute coercion as it removes an option that one is entitled to, that is, non-vaccination with the “Pay”. So too is the Italian scheme of fines coercive.

However, paying people is not coercive. Adding an option, like payment, without affecting the status quo is not coercive. If a person chooses that option, it is because they believe that overall their life will go better with it, in this case, with the vaccination and the payment. The gamble may not pay off: some risk might eventuate and then it wasn’t worth it. But that is life—and probability.

It is true that the value of the option might exercise force over our rational capacities, but that is no different from offering a lot of money to attract a favoured job applicant.

What can be problematic about offers is exploitation. Exploitation exists where one offers less than a fair deal and a person only accepts it because of vulnerability from background injustice.

There are two ways to prevent exploitation. First, we can correct any background injustice that might cause it. In this case, the person would have little reason to accept the offer. Second, we can pay a fair minimum price for risk, as when we pay construction workers danger money. Paradoxically, this requires paying more, rather than less.^40^

But there is an important additional feature of vaccination. If a vaccine were deemed to be safe enough to offer on a voluntary basis without payment, it must be safe enough to incentivise with payment because the risks are reasonable. It may be that those who are poorer may be more inclined to take the money and the risk, but this applies to all risky or unpleasant jobs in a market economy. It is not necessarily exploitation if there are protections in place such as a minimum wage or a fair price is paid to take on risk.

So payment for vaccination which passes independent safety standards (and could reasonably be offered without payment) is not exploitation, if the payment is adequate.

### Undue influence?

A related concern is undue influence. Undue influence means that because of the attractiveness of the offer, I can’t autonomously and rationally weigh up the risks and benefits. It is sometimes understood as “were it not for the money, he would not do it”.

But that formulation is too broad—were it not for the money, many people would not go to work. Rather what the concept of ‘undue influence’ intends to capture is that the offer, usually money, bedazzles a person so that he or she makes a mistake in weighing up the risks and benefits. Someone offers Jones a million dollars to take on a risk of 99.99% of dying in a dangerous experiment. He just focuses on the money and takes a deal which is unfair and unreasonable. However, taking such an offer might be rational. If Jones’ daughter is about to die without a million dollars and he values her life more than his own, it might be both autonomous and rational to take the deal.

Because we cannot get into people’s minds, it is difficult in practice to unravel whether undue influence is occurring—how can you differentiate it from a rational decision? In practice, if it would be acceptable to be vaccinated for nothing, it is acceptable to do it for money. Concerns about undue influence are best met by implementing procedures to minimise bias and irrational decision making, such as cooling off periods, information reframing, and so on.

There remains a lurking concern that a decision where payment is involved may not be fully autonomous or authentic. For example, racial and ethnic minorities are among the groups most gravely affected by COVID-19, but given concerns about systemic racism in research and medicine, these communities may have good reason to distrust the medical machine. Is it acceptable to use payment to get over those concerns?

All we can do practically to address concerns about autonomy and authenticity is to redouble efforts: to ensure we do know the risks and they are reasonable (and that the underpinning research is not itself subject to concerns about systemic racism), and try to foster trust with such public education campaigns. This can work alongside a payment scheme. People still need to understand what the facts are. They still need to make as autonomous and authentic a decision as possible.

### Practical advantages

A payment model could also be superior to a mandatory model from a practical point of view. There may be considerable resistance to a mandatory model which may make it difficult, expensive and time-consuming to implement, with considerable invasion of liberty. In a payment model, people are doing what they want to do.

A payment model could also be very cheap, compared with the alternatives. The cost of the UK’s furlough scheme is estimated to reach £60 billion by its planned end in October,^41^ and the economic shut down is likely to cost many billions more, as well as the estimated 200 000 lives expected to be lost as a result.^11^ It would make economic sense to pay people quite a lot to incentivise them to vaccinate sooner rather than later—which, for example, would speed up their full return to work.

It may be that payment is only required to incentivise certain groups. For example, it may be that young people require incentivising because they are at lower risk from the disease itself. On the other hand, justice might require payment for all taking the risk. Although the elderly and those at higher risk have more to gain personally, they are also providing a service by being vaccinated and not using limited health resources. (There is an enormous backlog of patients in the NHS—another grave threat to public health.)

One particularly difficult case is paying parents to vaccinate their children. It is one thing to pay people to take on risk for themselves; it is quite another to pay them to enable their children to take on risks, particularly when the children have little to gain as they are at lowest risk. In part, the answer to this issue is determined by how safe the vaccine is and how confident we can be in that assessment. If it were safe, to a level that even a mandatory programme would be justified, it may be appropriate to instead incentivise parents to volunteer their children for vaccination. If safety is less certain, payment for risk in this group is the most problematic.

It is true that some mandatory vaccination programmes already fine parents for failure to vaccinate their children. However, in those cases vaccination is clearly in the child’s best interest, as the child receives the benefit of immunity to diseases such as measles, that pose a greater risk to that child than we currently believe COVID-19 does. Moreover, they are for vaccines that have been in place for many years and have a well-established safety profile.

### Solidarity

A standard objection to paying people to do their duty, particularly civic duty, is that it undermines solidarity, trust, reciprocity and other community values. This is the argument given by Richard Titmuss for a voluntary blood donation scheme.^42^

The UK does not pay donors for blood or blood products, but does purchase blood products from other countries, including Austria where donors are paid a “travel allowance” for plasma donation. In Australia, which runs a donor system, more than 50% of the plasma comes from paid donors in the USA.^43^ Altruism is insufficient. Germany recently moved to paying for plasma donors. It does not appear to have undermined German society.

In the end, the policy we should adopt towards COVID-19 vaccination will depend on the precise risks and benefits of the vaccine (and our confidence in them), the state of the pandemic, the nature of the alternatives, and particularly the public appetite for a vaccine.

In the right circumstances, mandatory vaccination could be ethically justified, if the penalty is suitably proportionate.

Payment for vaccination, perhaps, has even more to be said for it.

For those attached to the gift of altruism, the vaccinated could be offered the opportunity to donate their fee back to the NHS (or similar health service provider). This combined “payment-donation” model would be a happy marriage of ethics and economics. It would give altruists a double chance to be altruistic: first by vaccinating and second by donating the fee. It would also couple self-interest with morality for free-riders (as they would have greater self-interest to do what is moral), and it would give those who face obstacles to vaccination an additional reason to overcome these.

### Payment in kind

Of course, benefits can come in cash or kind. An alternative “payment” model is to pay those who vaccinate in kind. This could take the form of greater freedom to travel, opportunity to work or socialise. With some colleagues, I have given similar arguments in favour of immunity passports.^44^

One attractive benefit would be the freedom to not wear a mask in public places if you carried a vaccination certificate, and not to socially distance. Currently, everyone has to wear a mask and practise social distancing. Relaxing this requirement for those who have been vaccinated (or otherwise have immunity) would be an attractive benefit. Moreover, it would help ameliorate the risks the unvaccinated would pose to others.

Payment in kind has one advantage over cash in that it might not send the signal that vaccination is perceived to be unsafe. A cash payment may paradoxically undermine vaccination uptake by introducing unwarranted suspicion (though this is an intuition that may need to be tested). Benefits in kind are less susceptible to this concern because they are directly linked to the benefit provided by the vaccine itself: the vaccinated person is no longer a threat to others.

Some might object that this represents a form of shaming the non-vaccinators (some of whom might be excluded from vaccination for health reasons), just as presenting those who evaded conscription with a white feather was a method of shaming perceived free-riders. But this could be managed through an education campaign about the justification for face covering requirements. There is a good reason to require the non-vaccinated to continue to wear masks and practice social distancing, regardless of whether their refusal is justified—they do represent a greater direct threat to others.

It is quite possible that some mixture of altruism, financial and non-financial benefits will obviate the need to introduce mandatory vaccination. It is better that people voluntarily choose on the basis of reasons to act well, rather than being forced to do so. Structuring the rewards and punishments in a just and fair way is one way of giving people reasons for action.

Mandatory vaccination can be ethically justified (see figure 3), but when risks are more uncertain, payment for vaccination (whether in cash or kind) may be an ethically superior option.
